# Does PRRT with standard activities of ^177^Lu-octreotate really achieve relevant somatostatin receptor saturation in target tumor lesions?: insights from intra-therapeutic receptor imaging in patients with metastatic gastroenteropancreatic neuroendocrine tumors

**DOI:** 10.1186/2191-219X-3-82

**Published:** 2013-12-26

**Authors:** Amir Sabet, James Nagarajah, Ahmet Semih Dogan, Hans-Jürgen Biersack, Amin Sabet, Stefan Guhlke, Samer Ezziddin

**Affiliations:** 1 Department of Nuclear Medicine, University Hospital, Sigmund-Freud-Str. 25, Bonn 53105, Germany; 2 Department of Nuclear Medicine, University Hospital, Essen 45122, Germany

## Abstract

**Background:**

Peptide receptor radionuclide therapy (PRRT) with ^177^Lu-[DOTA^0^,Tyr^3^]octreotate (^177^Lu-octreotate) is generally performed using a fixed activity of 7.4 GBq (200 mCi) per course bound to 180 to 300 μg of the peptide. While this single activity may lead to suboptimal radiation doses in neuroendocrine tumors (NET) with advanced or bulky disease, dose escalation has been withheld due to concerns on potential tumor somatostatin receptor saturation with reduced efficacy of the added activity. *In vivo* saturation effects during standard-dose PRRT based on quantification of pre- and intra-therapeutic ^68^Ga-DOTATOC positron emission tomography (PET) imaging might guide potential dose escalation.

**Methods:**

Five patients with metastatic NET of the pancreas underwent ^68^Ga-DOTATOC PET/CT before and directly after standard-dose PRRT with ^177^Lu-octreotate. In each patient, four target tumor lesions, normal liver parenchyma, and the spleen were evaluated and the ratios of SUV_max_ of the target lesions to liver (SUV_T/L_) and spleen (SUV_T/S_) were calculated; paired Student's *t* test was performed with *p* < 0.05 for pre-/intra-PRRT comparisons.

**Results:**

The mean intra-therapeutic tumor SUV_max_ showed no significant change (per-lesion paired *t* test) compared to pretreatment values (-9.1%, *p* = 0.226). In contrast, the SUV_max_ of the normal liver parenchyma and spleen were significantly lower directly after infusion of 7.4 GBq ^177^Lu-octreotate. Consequently, SUV_T/L_ and SUV_T/S_ increased significantly from pretreatment to intra-therapeutic examination: SUV_T/L_ (*p* < 0.001) from 2.8 ± 1.3 (1.3 to 5.8) to 4.7 ± 3.0 (2.1 to 12.7) and SUV_T/S_ (*p* < 0.001) from 1.2 ± 0.7 (0.4 to 3.0) to 3.5 ± 1.5 (1.6 to 7.9).

**Conclusions:**

This small retrospective study provides preliminary evidence for the absence of relevant *in vivo* saturation of somatostatin receptor subtype 2 (sst2) in tumor lesions during PRRT with standard activities of ^177^Lu-octreotate in contrast to normal tissue (liver, spleen) showing limited receptor capacity. After being confirmed by larger series, this observation will have significant implications for PRRT: (1) Higher activities of ^177^Lu-octreotate might be considered feasible in patients with high tumor disease burden or clinical need for remission, and (2) striving to reduce the amount of peptide used in standard preparations of ^177^Lu-octreotate appears futile.

## Background

Neuroendocrine tumors (NET) commonly overexpress somatostatin receptors, in particular the subtype 2 (sst2), which are targeted for tumor-directed imaging and therapy [1-4]. ^68^Ga-labeled somatostatin analogues with high affinity to sst2 such as ^68^Ga-DOTA-d-Phe^1^-Tyr^3^-octreotate or ^68^Ga-DOTA-d-Phe^1^-Tyr^3^-octreotide are widely used for positron emission tomography (PET) imaging of NET [5-7]. The same sst2 ligands coupled to β-emitters ^90^Y or ^177^Lu are successfully utilized for targeted radionuclide therapy, peptide receptor radionuclide therapy (PRRT) comprising a well-established, effective systemic treatment modality in patients with inoperable, metastatic gastroenteropancreatic NET (GEP NET) [8-11].

Standard PRRT with ^177^Lu-[DOTA^0^,Tyr^3^]octreotate (^177^Lu-octreotate) is performed with administration of a fixed activity of 7.4 GBq (200 mCi) per course [12-15]. This amount of activity is usually bound to 180 to 300 μg of the peptide with the chelator DOTA, [DOTA^0^,Tyr^3^]octreotate. One argument against dose escalation has been the concern on receptor saturation effects in the tumor lesions leading to reduced uptake and efficacy of the added activity [12,16-18]. This assumption has left PRRT with ^177^Lu-octreotate with an upper limit of activity per cycle of 7.4 GBq that until date has not been challenged; to the best of our knowledge, there are no reports of treatment with higher activities. However, it can be expected that this will lead to suboptimal treatment in some patients with more advanced/bulky tumor disease and a clinical need for tumor remission. The objective of this retrospective study was to investigate potential *in vivo* saturation of somatostatin receptors observed during standard PRRT according to quantification of intra-treatment PET studies.

## Methods

### Patients

The local committee on ethics approved this retrospective study, and all subjects had provided prior written informed consent. Pre- and post-treatment ^68^Ga-DOTATOC PET/CT images of five patients (three women and two men; age range, 47 to 79 years) with histologically confirmed, unresectable, metastatic NET of the pancreas (P-NET) who underwent one cycle of PRRT with ^177^Lu-octreotate between 2011 and 2012 were studied. All patients fulfilled the general inclusion criteria for PRRT including sufficient tumor uptake (i.e., ≥normal liver uptake) on baseline receptor imaging (^68^Ga-DOTATOC PET/CT) [19-21].

### PRRT

PRRT was performed with 7.4 GBq (200 mCi) ^177^Lu-octreotate administered in 30 min according to the standard protocol from Rotterdam. The ^177^Lu (IDB Holland, Baarle-Nassau, Netherlands) had a specific activity in the approximate range of 100 to 160 GBq/μmol at the time of administration. The peptide labeling [22,23] was performed such that an apparent specific activity of about 54 GBq/μmol (ratio of activity to the total amount of peptide) was obtained. Positively charged amino acids were coadministered for nephroprotection [12,24] (lysine 2.5% and arginine 2.5% in 1 L 0.9% NaCl; infusion of 250 mL/h).

### Somatostatin receptor PET imaging

DOTATOC was labeled with ^68^Ga eluted from an in-house ^68^Ge/^68^Ga generator following the procedure described by Zhernosekov et al. [25]. All patients underwent pretreatment as well as intra-therapeutic ^68^Ga-DOTATOC PET/CT: Pretreatment PET/CT was performed 1 to 3 days before PRRT and intra-therapeutic PET/CT during PRRT, with PET tracer injection 30 min after the start of the treatment infusion. To minimize the interfering effects of the time lag between treatment administration and PET tracer injection on quantitative analysis of intra-therapeutic images, ^68^Ga-DOTATOC was injected on the treatment ward without delay; the time interval between the end of ^177^Lu-octreotate infusion and ^68^Ga-DOTATOC injection for intra-therapeutic PET was <1 min. The scans were acquired from the base of the skull to the upper thighs (five to seven bed positions) 30 min after the injection of 200 MBq ^68^Ga-DOTATOC. The hybrid PET/CT scanner (Biograph 2, Siemens Medical Solutions Inc., Hoffman Estates, IL, USA) consisted of a dual-detector helical CT and a high-resolution PET scanner with a 16.2-cm axial field of view and a lutetium oxyorthosilicate (LSO) crystal detector (6.45 × 6.45 × 25 mm). CT was performed for attenuation correction and anatomical localization using the following parameters: 60 mAs, 130 kV, 0.8 s/tube rotation, slice thickness 5 mm, slice width 5 mm, and table feed 8 mm/s. Immediately following the CT image acquisition, PET data were acquired for 5 min per bed position. The coincidence time resolution was 500 ps with a coincidence window of 4.5 ns. The sensitivity was 5.7 cps/kBq at 400 keV. The attenuation-corrected PET data were reconstructed using a standardized ordered-subset expectation maximization (OSEM) iterative reconstruction with two iterations and eight subsets and a 5-mm Gaussian filter.

### Image analysis and statistical methods

The standardized uptake value (SUV) was determined as a measure of DOTATOC uptake. In each patient, three liver metastases with unimpaired delineation from other sources of tracer accumulation along with the primary tumor or an extrahepatic metastasis if the primary was resected were selected as target lesions, and normal liver parenchyma as well as the spleen (if not affected by tumor infiltration) as background control. Irregular regions of interest with a threshold of 50% of the SUV_max_ were drawn and the respective SUVs were recorded. In order to normalize tumor SUVs, the ratios of SUV_max_ of the target lesions to maximal hepatic uptake (SUV_T/L_) and maximal splenic uptake (SUV_T/S_) were calculated in pre- and post-treatment ^68^Ga-DOTATOC PET/CT images. Paired Student's *t* test was performed with a significance level of *p* < 0.05 to examine the changes in receptor status after administration of ^177^Lu-octreotate. The statistical software package SPSS (version 20, SPSS Inc., Chicago, IL, USA) was used to analyze the data.

## Results

In each of the five patients, four target tumor lesions, one normal liver region, and the spleen were evaluated. The mean SUV_max_ before therapeutic administration of ^177^Lu-octreotate was 22.5 ± 8.5 (range, 9.7 to 38.0) in the tumor lesions, 8.5 ± 2.5 (range, 6.5 to 11.6) in the normal liver parenchyma, and 21.4 ± 8.9 (range 11.4 to 31.7) in the spleen (Table 1). The highest intra-individual variation of SUV_max_ in the tumor lesions at baseline was 8.8 ± 5.7 (range 2.6 to 15.9).

**Table 1:** **SUV parameters of **^**68**^**Ga-DOTATOC PET imaging before and directly after administration of PRRT with 7.4 GBq **^**177**^**Lu-octreotate**

	**SUV**_**max **_**(mean ± SD)**		***p *** **value**^**a**^
	**Pretreatment**	**Intra-therapeutic**	
Tumor lesions	22.5 ± 8.5	20.0 ± 10.4	0.226
Normal liver	8.5 ± 2.5	4.8 ± 2.0	0.016
Spleen	21.4 ± 8.9	5.6 ± 0.8	0.015
SUV ratio			
Tumor-to-liver	2.8 ± 1.3	4.7 ± 3.0	<0.001
Tumor-to-spleen	1.2 ± 0.7	3.5 ± 1.5	<0.001

^a^Paired *t* test.

**Figure 1:**
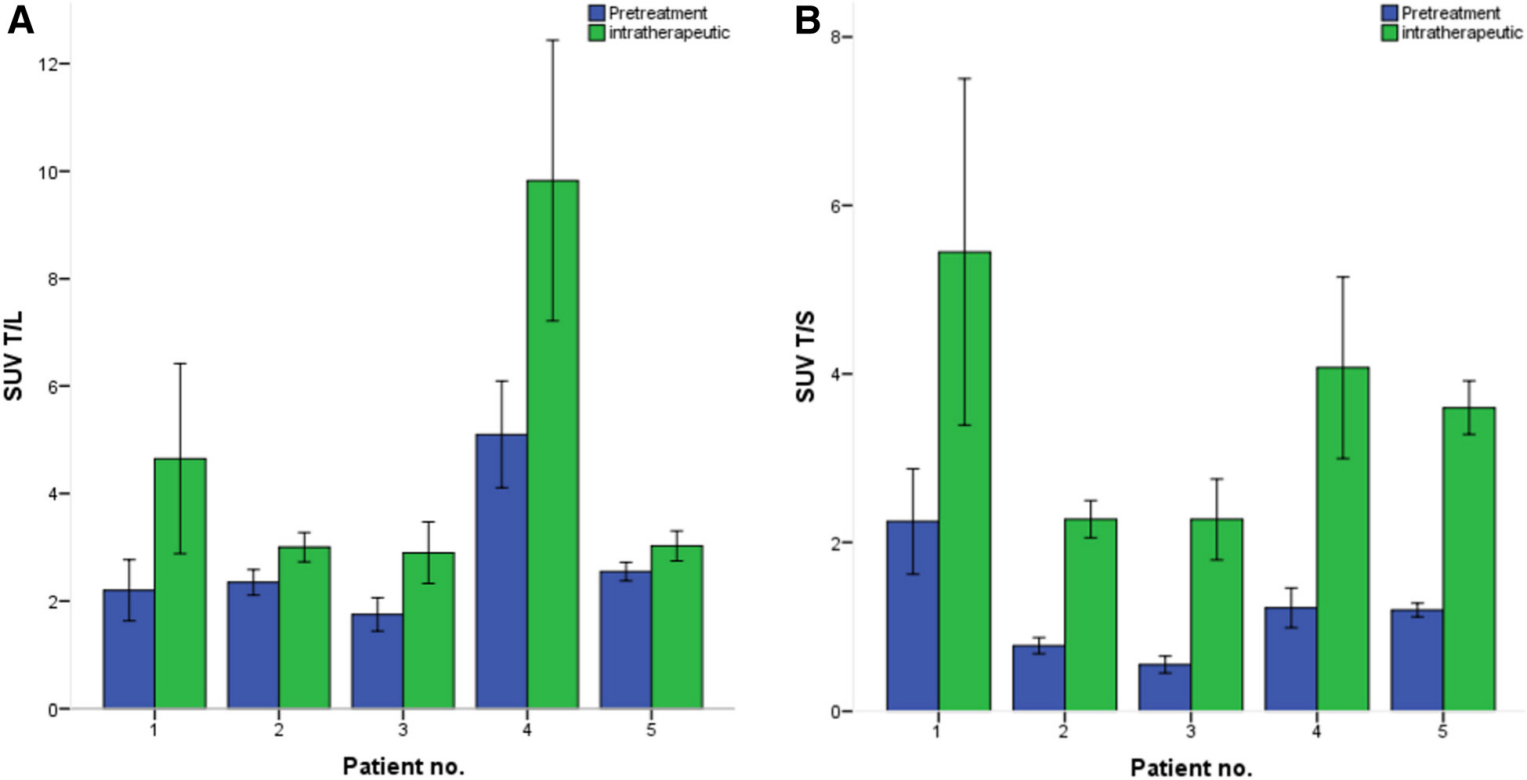
**SUV ratios of target lesions to normal tissue in pre- and intra-therapeutic settings using ^68^Ga-DOTATOC PET.** The images show the comparison of the mean tumor-to-liver **(A)** and tumor-to-spleen **(B)** SUV ratios in each patient (*n* = 5).

**Figure 2:**
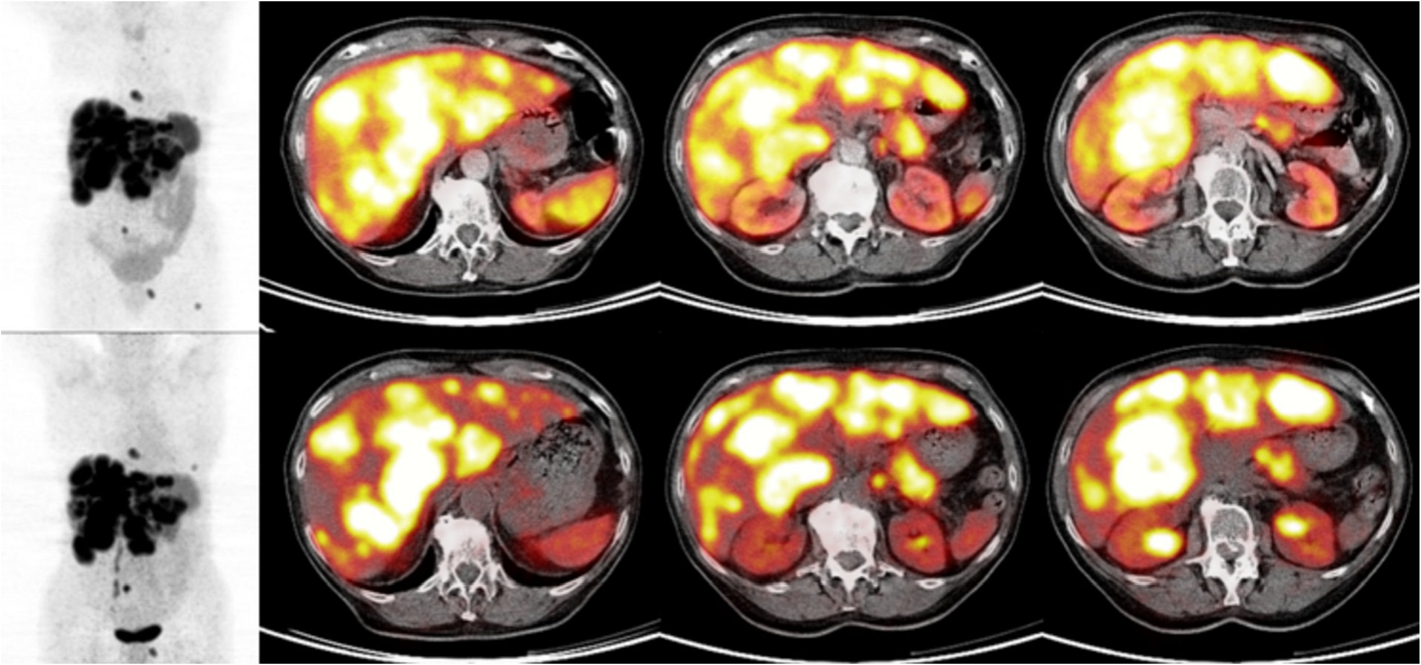
**Pretreatment (above) and intra-therapeutic (below) ^68^Ga-DOTATOC PET/CT images of a patient.** The patient has liver and bone metastases of a P-NET (tail of the pancreas) and high hepatic tumor disease burden. The significant increase of tumor-to-nontumor SUV ratios (SUV_T/L_ and SUV_T/S_) after ^177^Lu-octreotate administration (+111.4% and +142.2%, respectively, both *p* < 0.001) is visible on the images.

Directly after therapeutic infusion of 7.4 GBq ^177^Lu-octreotate, the mean 'intra-therapeutic’ tumor SUV_max_ of injected ^68^Ga-DOTATOC was 20.0 ± 10.4 (range, 7.6 to 50.7), showing no significant change on per-lesion comparison (-9.1%; paired *t* test, *p* = 0.226). In contrast, the intra-therapeutic SUV_max_ of the normal liver parenchyma (4.8 ± 2.0; range, 2.3 to 7.5) and the spleen (5.6 ± 0.8; range, 4.7 to 6.4) were significantly lower compared to pretreatment values (*p* = 0.016 and *p* = 0.015, respectively). Consequently, the tumor-to-nontumor ratios (SUV_T/L_ and SUV_T/S_) increased significantly from pretreatment to intra-therapeutic assessment: SUV_T/L_ (*p* < 0.001) from 2.8 ± 1.3 (1.3 to 5.8) to 4.7 ± 3.0 (2.1 to 12.7) and SUV_T/S_ (*p* < 0.001) from 1.2 ± 0.7 (0.4 to 3.0) to 3.5 ± 1.5 (1.6 to 7.9). Figures 1 and 2 illustrate the mean change of SUV_T/L_ and SUV_T/S_ in each patient and in a sample patient, respectively.

## Discussion

This small retrospective study with 20 analyzed tumor lesions in five patients provides preliminary evidence for the absence of relevant *in vivo* receptor saturation effects regarding the targeted sst2 receptors in tumor lesions during PRRT with standard activities of ^177^Lu-octreotate. This observation is contrasted by highly significant saturation effects in normal (nontumor) tissue depicted by somatostatin receptor PET imaging.

Binding of labeled or unlabeled somatostatin analogues to the highly overexpressed membranous sst2 in NET triggers a fast and efficient internalization process of the ligand-receptor complex within 2.5 min [26]. In nuclear medicine, this mechanism results in very effective accumulation of intra-cellular radiolabeled peptides and permits successful tumor imaging and targeted radionuclide therapy [27-29]. The 'recycling’ process of the internalized receptors to the plasma membrane is, on the other hand, relatively slow and may take several hours [26]. This results in saturation-like effects whenever infusion of radiolabeled sst2 ligands is preceded by application of labeled or unlabeled ligands and limits the amount of radiolabeled molecules and thus activity to be delivered into the tumor. The ^68^Ga-DOTATOC uptake in intra-therapeutic PET imaging directly after ^177^Lu-octreotate administration, i.e., 30 min after the beginning of the radiopeptide infusion, is mediated by the 'unsaturated’ membranous sst2 binding the PET tracer compound; the extent of its uptake is probably not influenced by (if at all present) recently recycled receptors after ^177^Lu-octreotate infusion. This imaging approach may allow effective *in vivo* quantification of the (unbound and available) membranous receptor density during PRRT.

Few studies have reported the possible positive effects associated with the presence of high peptide amounts on the uptake of radiolabeled somatostatin analogues in tumor tissues [28,30-32]. A systematic investigation of the impact of peptide mass on the uptake of ^68^Ga-DOTATOC in NET revealed a saturable tracer accumulation in normal organs (spleen and liver) after the administration of 50 μg of unlabeled octreotide [31]. In contrast to this, a saturation effect in the tumor tissue was observed only after preloading with considerably higher amounts of unlabeled octreotide (250 or 500 μg). The increase of selective uptake in tumor lesions after injection of 50 μg octreotide in the same study moreover indicated potential up-regulation of tumor somatostatin receptor density by unlabeled octreotide [31]. Similar results have been observed after administration of long-acting somatostatin 14.5 to 11.4 days prior to PET/CT examination [30]. To date, however, radionuclides with high specific activity and relatively low amounts of peptide are suggested for the purpose of PRRT. Consequently, treatment with ^177^Lu-octreotate is performed with up to 7.4 GBq ^177^Lu-octreotate to restrict the peptide mass and avoid somatostatin saturation in tumor lesions. The results of our study confirm the absence of relevant receptor saturation in tumor lesions, while at the same time demonstrating a striking saturation in the spleen and liver, during standard treatment with 7.4 GBq ^177^Lu-octreotate; this finding may encourage dose escalation strategies, such as in patients with higher tumor disease burden.

Application of different somatostatin analogues, octreotide (^68^Ga-DOTATOC) and octreotate (^177^Lu-[DOTA^0^,Tyr^3^]octreotate), for imaging and treatment may be regarded as one limitation in this study. Considering the predominant affinity of these somatostatin analogues for the same receptor subtype, the sst2, the receptor 'saturation’ after treatment with ^177^Lu-octreotate may very well be determined using ^68^Ga-DOTATOC PET. The SUV_max_ values are in line with reported results of other authors on ^68^Ga-DOTATOC PET [33,34]. The main limitation of our study is the small population size (*n* = 5), which obviously restricts the strength of our conclusions. However, the striking and highly significant increase of tumor-to-background ratios of ^68^Ga-DOTATOC uptake (SUV_T/L_ and SUV_T/S_) after ^177^Lu-octreotate administration with no significant change in the uptake intensity of target lesions (*n* = 20) clearly indicates receptor saturation of normal tissues with at the same time preserved receptor capacity of tumor lesions during standard treatment with 7.4 GBq ^177^Lu-octreotate.

## Conclusions

Derived from a small series of intra-individual comparison of pre- and intra-therapeutic somatostatin receptor PET imaging, there is no evidence for clinically relevant somatostatin receptor saturation of targeted tumor lesions. For PRRT with standard activities, the tumor receptor capacity of sst2 does not seem to be nearly reached in contrast to the obviously limited receptor-mediated radiopeptide uptake capacity of normal tissue such as the liver and spleen. After being confirmed by larger series, this observation may have important implications for PRRT, in that (1) higher activities of ^177^Lu-octreotate (>7.4 GBq) might be considered whenever high tumor disease burden or clinical need for remission exceeds toxicity issues and (2) the amount of peptide used in standard preparations of ^177^Lu-octreotate is viewed as uncritical, i.e. attempts to reduce peptide mass by employing ^177^Lu of ultrahigh specific activity would appear nonbeneficial.

## Competing interests

The authors declare that they have no competing interests.

## Authors' contributions

AS drafted the manuscript and contributed to the data collection. JN contributed in the analysis and interpretation of the data and drafting of the manuscript. ASD collected the data and drafted the figures and tables. HJB participated in the design and coordination of the study. AS drafted the figures and tables and contributed in the collection of data and manuscript editing. SG contributed to the concept of the study and critical revision of the article. SE conceived of the study concept, interpreted the data, and determined the methodology and directions of manuscript drafting including discussion of results. All authors read and approved the final manuscript.
